# Cell type matching across species using protein embeddings and transfer learning

**DOI:** 10.1093/bioinformatics/btad248

**Published:** 2023-06-30

**Authors:** Kirti Biharie, Lieke Michielsen, Marcel J T Reinders, Ahmed Mahfouz

**Affiliations:** Delft Bioinformatics Lab, Delft University of Technology, Delft 2628XE, The Netherlands; Department of Human Genetics, Leiden University Medical Center, Leiden 2333ZC, The Netherlands; Leiden Computational Biology Center, Leiden University Medical Center, Leiden 2333ZC, The Netherlands; Delft Bioinformatics Lab, Delft University of Technology, Delft 2628XE, The Netherlands; Department of Human Genetics, Leiden University Medical Center, Leiden 2333ZC, The Netherlands; Leiden Computational Biology Center, Leiden University Medical Center, Leiden 2333ZC, The Netherlands; Delft Bioinformatics Lab, Delft University of Technology, Delft 2628XE, The Netherlands; Department of Human Genetics, Leiden University Medical Center, Leiden 2333ZC, The Netherlands; Leiden Computational Biology Center, Leiden University Medical Center, Leiden 2333ZC, The Netherlands; Delft Bioinformatics Lab, Delft University of Technology, Delft 2628XE, The Netherlands; Department of Human Genetics, Leiden University Medical Center, Leiden 2333ZC, The Netherlands; Leiden Computational Biology Center, Leiden University Medical Center, Leiden 2333ZC, The Netherlands

## Abstract

**Motivation:**

Knowing the relation between cell types is crucial for translating experimental results from mice to humans. Establishing cell type matches, however, is hindered by the biological differences between the species. A substantial amount of evolutionary information between genes that could be used to align the species is discarded by most of the current methods since they only use one-to-one orthologous genes. Some methods try to retain the information by explicitly including the relation between genes, however, not without caveats.

**Results:**

In this work, we present a model to transfer and align cell types in cross-species analysis (TACTiCS). First, TACTiCS uses a natural language processing model to match genes using their protein sequences. Next, TACTiCS employs a neural network to classify cell types within a species. Afterward, TACTiCS uses transfer learning to propagate cell type labels between species. We applied TACTiCS on scRNA-seq data of the primary motor cortex of human, mouse, and marmoset. Our model can accurately match and align cell types on these datasets. Moreover, our model outperforms Seurat and the state-of-the-art method SAMap. Finally, we show that our gene matching method results in better cell type matches than BLAST in our model.

**Availability and implementation:**

The implementation is available on GitHub (https://github.com/kbiharie/TACTiCS). The preprocessed datasets and trained models can be downloaded from Zenodo (https://doi.org/10.5281/zenodo.7582460).

## 1 Introduction

Model organisms, such as mouse and marmoset, are often used in brain research as a substitute for humans. However, because of differences between species, experiments performed on model organisms do not directly translate to humans. For example, widely used antidepressants that target serotonin receptors are often tested on mice, while the expression pattern of serotonin receptors is highly divergent between human and mouse, likely leading to differences in cell function between species (Hodge et al. 2019). Consequently, to facilitate translational research, it is important to better characterize cell type matches between species. This facilitates studying how drugs then alter biological processes within specific cell types between these species.

Traditionally, cell types were characterized solely based on morphology, but using single-cell RNA sequencing (scRNA-seq), the expression pattern across thousands of genes can now be used to describe a cell type. This has resulted in the identification of an increasing number of cell types within specific brain regions (Tasic et al. 2018; Siletti et al. 2022). Although this improves our understanding of biological processes in the brain, when comparing species, it introduces the need for a method that can match these new cell types accurately between species.

Unfortunately, this is not a trivial task as genes are modified, duplicated and deleted throughout evolution, resulting in complicated many-to-many gene–gene relationships between species. These relationships become even more complicated when evolutionary distances increase.

Current methods that match cell types across species based on scRNA-seq data can be divided into two groups, mainly based on how they solve the gene-matching problem. The first group only uses the one-to-one orthologous genes, which are genes with exactly one match in the other species based on sequence similarity [e.g. using BLAST (Altschul et al. 1990)]. Methods such as scANVI (Xu et al. 2021), MetaNeighbour (Crow et al. 2018), and LAMbDA (Johnson et al. 2019) belong to this group. While this is a straightforward approach, it ignores genes with a more complex evolutionary history which might have caused divergent functional specification of cell types between species. The second group of methods, including SAMap (Tarashansky et al. 2021), CAME (Liu et al. 2021), Kmermaid (Botvinnik et al. 2021), and C3 (Kabir et al. 2018), overcomes this limitation by considering many-to-many relationships between the genes based on sequence similarity. All these methods rely on the classical assumption that sequence similarity is a good measure of how genes functionally relate to each other. However, sequence similarity often considers one nucleotide/amino acid at a time, which to a large extent ignores sequence contexts important for functional characterization (e.g. secondary structures and sequence motifs). A growing body of evidence suggests that language models are a powerful approach to capture functional similarities between genes (Heinzinger et al. 2019; Elnaggar et al. 2021; Rives et al. 2021; Villegas-Morcillo et al. 2021). Similarly, we hypothesize that using language models to match genes between species can be beneficial for cell type matching.

Once we identified matching relationships between genes across species, the next step is to characterize cell type matches. We and others have posed cell type matching as a classification task where the agreement of predictions from two classifiers, trained on two labeled scRNA-seq datasets, is used to match cell types between the datasets (Johnson et al. 2019; Michielsen et al. 2021; Yuan et al. 2022). Biological differences between species, however, hinder applying such a method directly. A solution could be to learn a common embedding space for the cells before training the classifiers.

Here, we introduce a method to transfer and align cell types in cross-species analysis (TACTiCS) that incorporates the two claims that we make: (1) using language models to match genes functionally between species and (2) training classifiers in a shared embedding space to transfer cell types from one species to the other. We show that TACTiCS correctly matches human, mouse, and marmoset brain cell populations from the primary motor (M1) cortex at a detailed cell type level, and does so better than SAMap, the current state-of-the-art method.

## 2 Methods

**Figure 1.**
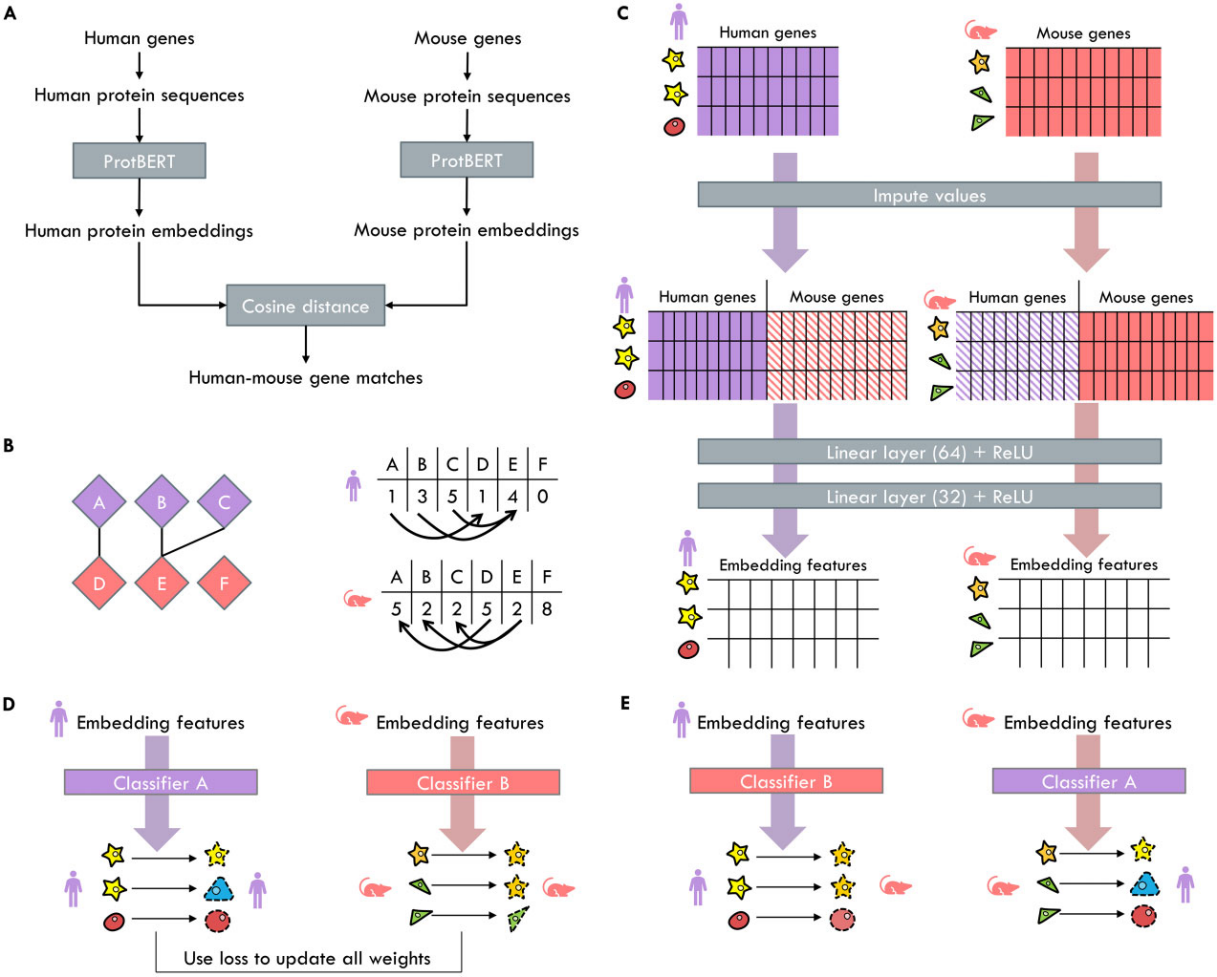
Schematic overview of TACTiCS. We use human and mouse as example, but cell types from any two species can be matched. (A) Matching genes on protein sequences using ProtBERT. (B) Bipartite graph of gene matches. Gene expression is imputed by taking the weighted average from connected genes in the bipartite graph. (C) Creating cell embeddings using linear layers on the shared feature space. The weights of the linear layers are shared. (D) Classifying within-species cells during training. The classifier consists of a linear layer outputting the cell type probabilities followed by a softmax. (E) Classifying cross-species cells using transfer learning. The predictions are used to match cell types.

TACTiCS takes as input two single-cell (sc) or single-nucleus (sn) RNA-seq datasets, with raw expression counts, from two species A and B. TACTiCS consists of four steps (Fig. 1): (1) matching genes based on the protein sequences, (2) creating a shared feature space by mapping expression values with the gene matches obtained in step 1, (3) training within-species cell type classifiers, and (4) matching cell types by swapping the classifiers.

### 2.1 Matching genes

First, we created an embedding for every gene using ProtBERT, a transformer-based language model (Elnaggar et al. 2021). The protein sequences were retrieved from UniProt (The UniProt Consortium 2023). For human and mouse, we selected only the Swiss-prot sequences, but for marmoset we selected all protein sequences. We input the protein sequences to ProtBERT to create an embedding for each protein $h^{\text{ProtBERT}}$ (Fig. 1A). ProtBERT generates a 1024-dimensional embedding for every amino acid in the protein sequence. To allow TACTiCS to work with variable-length proteins, we followed common practice (Heinzinger et al. 2019) and took the mean embedding over all positions to represent the whole protein sequence (as well as the corresponding gene). Protein sequences longer than 2500 amino acids (<2% of all sequences) were truncated to the first 2500 to fit into the memory of the GPU.

Next, for every pair of genes from species A and species B, we calculated the cosine distance between the ProtBERT embeddings. The initial set of gene matches were pairs with a cosine distance ≤0.005. To ensure that a gene is not connected to too many genes, we kept only the five closest genes, that met the distance threshold, for every gene.

Finally, we filtered the informative gene matches. Hereto, we calculated the top 2000 highly variable genes per species using Scanpy highly_variable_genes, and kept only those gene matches where at least one of the two genes is within the set of highly variable genes in their respective species (Wolf et al. 2018). From these matches, we constructed two sets of genes $G_{A}$ and $G_{B}$, corresponding to species A and B, respectively, consisting of genes with a match in the other species.

To obtain sequence similarity based gene matches, we used BLAST instead of ProtBERT. To obtain the many-to-many BLAST matches, we elected matches with an *E*-value <1*e*−6 as the initial set of matches. We used the bitscore as the distance metric. Since BLAST is not symmetrical, one gene match is assigned a separate *E*-value and bitscore for each direction. If only one direction meets the *E*-value threshold, we use the corresponding bitscore as the gene distance. If both directions meet the threshold, we use the average of the two bitscores. The list of matches is then filtered similarly as before with the closest-five and highly varying gene filtering. Additionally, we obtained one-to-one BLAST matches by starting with the same set of matches using the *E*-value threshold. For every gene, we kept only the best match, i.e. the gene with the highest bitscore. We discarded gene matches that were not reciprocal and finally also applied the highly varying gene filtering to obtain the one-to-one matches.

### 2.2 Creating a shared feature space by mapping expression values with the gene matches

We normalized the expression levels of genes as follows: (1) the raw expression counts of each dataset are normalized by the number of reads per cell such that the total number of counts in every cell is 10 000 and (2) the natural logarithm of the normalized counts are taken: where $x_{ij}$ is the expression of gene *j* in cell *i.* Finally, a *Z*-score per gene is calculated to form the normalized expression matrices $X^{A}$ and $X^{B}$ for genes $G_{A}$ and $G_{B}$, respectively. We created a shared feature space for the two datasets spanning $G_{A}\cup G_{B}$ (Fig. 1B). The shared feature space is partly equal to the expression matrices $X^{A}$ and $X^{B}$ and partly imputed: where $\bar{X_{iu}^{A}\;}$ is the normalized expression of cell i from species A for gene u in the shared feature space. The expression of within species genes does not change. For a cross-species gene, we imputed the expression by taking the weighted average of the expression of the within-species genes it is matched to. The weight between gene u and gene v is calculated as: where similarity calculates the cosine distance between the ProtBERT embeddings. The weights are scaled to the interval [0, 1] by dividing with the distance threshold. When BLAST is used instead, we used the (average) bitscore between the two genes directly, since the bitscore does not have to be inversed. The edge weight is set to 0 for gene pairs that do not match according to the threshold and filtering criteria. The resulting matrices $\bar{X^{A}}$ and $\bar{X^{B}}$ both span the same set of genes, and can thus be compared directly.


$$x_{ij}=\ln\left(\frac{x_{ij}}{\sum_{k\in G}x_{ik}\;}*{1e}^{5}+1\right),$$

$$\bar{X_{iu}^{A}\;}=\left\{\begin{array}{ll} X_{iu}^{A}, & \mathrm{if}\;u\in G_{A}\\ \frac{1}{\sum_{v\in G_{A}}e_{uv}}\sum_{v\in G_{A}}e_{uv}X_{iv}^{A}, & \mathrm{if}\;u\in G_{B} \end{array}\right.$$

$$e_{uv}=1-\frac{\mathrm{similarity}(h_{u}^{\text{ProtBERT}},h_{v}^{\text{ProtBERT}})}{0.005},$$

### 2.3 Cell embeddings

The shared feature space is put through two linear layers to create the cell embeddings (Fig. 1C). Each linear layer is followed by a rectified linear unit (ReLU) activation function. The first layer creates embeddings of length 64. The second layer creates embeddings of length 32. These embeddings are used to visualize the embedding space with a UMAP. The weights to embed the cells are shared across the species.

### 2.4 Training species-specific cell type classifier

We used these embeddings to train a separate classifier per species. We used a neural network consisting of one linear layer followed by a softmax activation function (Fig. 1D). Both classifiers take the cell embedding as input and output cell type probabilities, $h^{A,\mathrm{out}}$ or $h^{B,\mathrm{out}}$, only for cell types belonging to its respective species. During training, cells are input only to the classifier of its corresponding species.

The loss to update the embedding and classification weights consists of two parts: (1) the classification loss and (2) the alignment loss. Both losses are calculated separately per species. For the classification loss, we used the weighted cross-entropy loss between the predictions and targets: where $L_{\mathrm{cl}s_{A}}$ is the classification loss for species A. $N_{A}$ and $T_{A}$ are the number of cells and cell types in species A, respectively. $w_{t}$ is the weight for cell type t, explained further below. $h_{it}^{A,\mathrm{out}}$ is the output of classifier A, specifically the probability that cell i belongs to cell type t. The one-hot encoded targets Y are modified with label smoothing to prevent overfitting and improve stability: where ϵ (=0.1) controls the smoothness. The weight of each cell type is updated every epoch based on the accuracy of that cell type: where $\text{ac}c_{t}$ is the accuracy of class t in the current epoch. α is a hyperparameter that controls the influence of the accuracy on the weight. We use α=9 such that the weights are in the interval $\left[1,10\right]$ which restricts the relative difference in weight between cell types. By updating the weights, a cell type with a lower accuracy in the current epoch will have a higher weight in the next epoch and thus the predictions will shift to that cell type.


$$L_{\mathrm{cl}s_{A}}=\frac{1}{N^{A}}\sum_{i=1}^{N_{A}}\sum_{t=1}^{T_{A}}w_{t}Y_{it}^{\mathrm{LS}}\ln\left(h_{it}^{A,\mathrm{out}}\right)\;,$$

$$Y_{it}^{\mathrm{LS}}=\left\{\begin{array}{ll} 1-\epsilon , & \mathrm{if}\;Y_{i}=t\\ \frac{\epsilon }{T-1}, & \mathrm{otherwise} \end{array}\right.$$

$$w_{t}=\left(1-\text{ac}c_{t}\right)*\alpha +1,$$

The alignment loss aims to integrate the embedding space across the species, such that cross-species cells with a similar gene expression are close in the embedding space: where $N^{A}$ is the number of cells of species A and $N_{i}^{\mathrm{cross}}$ are the 20 nearest cross-species neighbours for cell i. MSE calculates the mean-squared error between the prediction of the shared features of neighbours j and the actual shared features for cell i. If the alignment loss is minimized, neighbours in the embedding space can be used to predict the gene expression. The final loss is a combination of the classifier loss, the alignment loss and a regularization loss: where θ consists of all parameters in the model, and is used for the L2 regularization to prevent overfitting. γ is the weight of the L2 norm, which is set to 0.01. The model is trained for 200 epochs. We used the Adam optimizer with a learning rate of 0.001. The full training process takes around 30 min.


$$L_{\mathrm{alig}n_{A}}=\;\frac{1}{N^{A}}\sum_{i=1}^{N^{A}}\text{MSE}\left(\frac{1}{\left|N_{i}^{\mathrm{cross}}\right|}\sum_{j\in N_{i}^{\mathrm{cross}}}\bar{X_{j}^{B}},\;\bar{X_{i}^{A}}\right),$$

$$L=L_{\mathrm{cl}s_{A}}\;+\;L_{\mathrm{cl}s_{B}}\;+\;L_{\mathrm{alig}n_{A}}\;+\;L_{\mathrm{alig}n_{B}}\;+\gamma {\left\|\theta \right\|}_{2}^{2},$$

To efficiently use large scRNA-seq datasets, the neural network is trained in batches. A batch size of 5000 cells per species is used to speed up the training while still having enough cells per cell type. Instead of sequentially iterating over the dataset, each batch is randomly sampled from the full dataset, while accounting for the size of each cell type. More specifically, every cell is assigned a probability $N^{A}/N_{t}^{A}$ or $N^{B}/N_{t}^{B}$, where $N^{A}$ is the total number of cells of species A and $N_{t}^{A}$ is the number of cells of species A belonging to cell type t. These probabilities are then used to sample a batch of cells per species with a similar number of cells for each cell type.

### 2.5 Transferring cell type predictions across species

After the neural network is trained, the cell types are transferred by using the classifiers on the species they were not trained on (Fig. 1E). That is, we calculate $h^{B,\mathrm{out}}\;$for cells of species A, and $h^{A,\mathrm{out}}$ for cells of species B. The transferred cell type for a single cell is the cell type with the highest probability. To aggregate the information of the single cells to the cell type, we calculate the fraction of cells that are predicted to match cell types across species, which forms a normalized confusion matrix for both transferring directions. We average the two matrices to create a combined matrix, where high values indicate reciprocal matches. The values in the combined matrix can be used to score a match.

### 2.6 Dataset

We evaluated TACTiCS on snRNA-seq data taken from the primary motor cortex of human, mouse, and marmoset (Bakken et al. 2021). These datasets consist of 76k human cells, 159k mouse cells, and 69k marmoset cells, respectively. The cell type distribution varies considerably across species. For instance, non-neuronal cells make up around a third of both mouse and marmoset cells, while only 5% of the human cells are non-neuronal. We use two resolutions of the cell labels assigned by the original authors: (1) a higher resolution, consisting of 45 cell types present in all species and (2) a lower resolution, consisting of 20 human, 23 mouse, and 22 marmoset subclass cell types. At the lower resolution not all cell types occur in all species. SMC is only present in mouse, while Meis2 and Peri are only present in mouse and marmoset. Species-specific cells are labeled with “NA” at the higher resolution.

### 2.7 Evaluation

The combined matrix cannot be evaluated using standard metrics for confusion matrices, such as precision or F1 score, since we cannot distinguish between false positives and false negatives. Instead, we focus on the matching scores from corresponding cell types in the combined matrix, which ideally should be 1. We define the average diagonal score (ADS) as the average score of the diagonal entries, after excluding species-specific cell types. A high ADS indicates that many cell types are correctly and reciprocally matched. However, the ADS does not indicate how many cell types are correctly matched. To this end, we define the recall as the fraction of diagonal entries where the score is highest for both that row and column.

We compared TACTiCS with SAMap (Tarashansky et al. 2021) and Seurat (version 4) (Hao et al. 2021). SAMap is a cell type matching method that iterates between two steps. The first step matches the genes, which is initially done with BLAST on the DNA or protein sequences. Instead of taking the top-1 match, SAMap uses the BLAST bitscore directly in their model which allows for many-to-many matches. The second step uses the gene matches to first impute genes across species and then embed the cells by concatenating the principal components of the original expression and imputed expression. Then, the correlation between genes in the embedding space is used to update the gene matches. The two steps are repeated until the process converges.

Seurat can be used to transfer cell type labels from a reference to a query dataset. Since Seurat cannot use many-to-many matches, we use BLAST one-to-one matches for the data integration and label transfer. Since labels can only be transferred from the reference to the query dataset, we had to integrate the data twice for each pairwise comparison: once using one species as the reference and once using the other species as the reference.

### 2.8 Implementation

TACTiCS is implemented in Python 3.9. Pytorch (Paszke et al. 2019) was used for the model architecture. The scRNA-seq data are stored as Anndata (Virshup et al. 2021) objects, containing both the gene expression and the cell type annotations. The implementation of TACTiCS is available at https://github.com/kbiharie/TACTiCS.

As Tarashansky et al. (2021) have noted, the runtime of SAMap increases significantly for larger datasets, and we were unable to run SAMap for the full datasets. Instead, we used SAMap on subsets of 50k cells per species. We subsampled the data to keep the cell type proportions similar while making sure that all cell types are included. During sampling we ensured that at least 50 cells were present in the subset. If a cell type contained less than 50 cells, all cells were included in the subset.

## 3 Results

### 3.1 Matching genes using sequence embeddings is comparable to sequence alignment with notable differences

First, we investigate how similar the gene matches returned by ProtBERT and BLAST are. We retrieved 17 435 human and 14 033 mouse protein sequences, discarding 47% of the human genes and 49% of the mouse genes for which we do not have the protein sequence. We used both ProtBERT and BLAST to generate gene matches.

**Figure 2.**
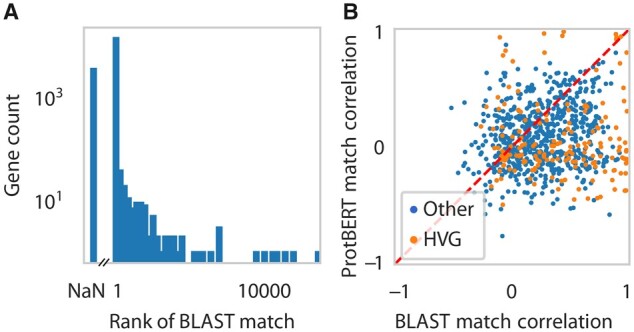
Comparison of ProtBERT and BLAST matches. (A) Rank of BLAST match according to ProtBERT embedding distances. Rank 1 indicates that the best ProtBERT match and the best BLAST match are the same. Rank NaN indicates a human gene with a ProtBERT match but no BLAST one-to-one match. (B) Scatterplot of the correlation of the expression of human and mouse genes when considering the best BLAST match (*x*-axis) and the best ProtBERT match (*y*-axis). The expression correlation is calculated as the Pearson correlation across the average expression profiles of the cross-species harmonized cell types. We omitted human genes where the BLAST match and ProtBERT match are the same. Gene matches where either the human gene, ProtBERT match or BLAST match is highly variable, are colored orange.

For 13 935 human genes, we found a one-to-one mouse match using BLAST. For these human genes, we defined the ProtBERT match as the mouse gene with the most similar ProtBERT embedding. For 13 050 of the 13 935 human genes (94%), the BLAST match is identical to the ProtBERT match. Thus, the top-1 match is identical for the vast majority of genes. We ranked the BLAST matches according to the ProtBERT embedding distance to all mouse genes (Fig. 2A). Most of the BLAST matches have a rank close to 1 and over 98% of the BLAST matches have a rank below 100. Additionally, 48% of the BLAST matches that differ from the ProtBERT match are in the top-5 and thus considered in the many-to-many matches. Thus, if the BLAST match is not considered to be the best match by ProtBERT, it is still relatively similar based on the embedding distance.

**Figure 3.**
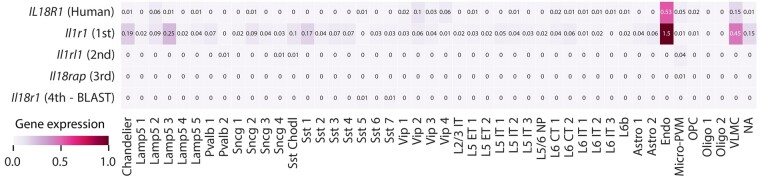
Average expression of human *IL18R1* and mouse matches across harmonized cell types. The mouse matches are ordered according to the ProtBERT embedding distances. BLAST matches human *IL18R1* to mouse *Il18r1*.

Next, we focus on the human genes for which the ProtBERT and BLAST match differ to investigate which method returns the most functionally similar match. We restrict the comparison to the 818 human genes where the human gene, the BLAST match and the ProtBERT match are expressed in at least one cell. We assess functional similarity here in terms of gene expression similarity across cell types. Therefore, we calculated the Pearson correlation coefficient across cell types in humans and mouse. We considered the harmonized cell types as defined in Bakken et al. (2021) (Fig. 2B). For 568 of 818 (69%) genes, the BLAST match has a higher gene correlation than the ProtBERT match. This is to be expected since the harmonized cell types were defined using the BLAST matches. However, for some genes, the ProtBERT match has a higher correlation than the BLAST match. For example, human *IL18R1* is matched to mouse *Il1r1* according to ProtBERT with a correlation coefficient of 0.945, while BLAST matches the gene to mouse *Il18r1* with a correlation coefficient of 0.103 (Fig. 3). Human *IL18R1* and mouse *Il1r1* both show an increased expression for the endothelial and VLMC cells, while mouse *Il18r1* does not show this pattern, and is lowly expressed in all cell types.

### 3.2 TACTiCS accurately matches cortical cell types across mouse and human

**Figure 4.**
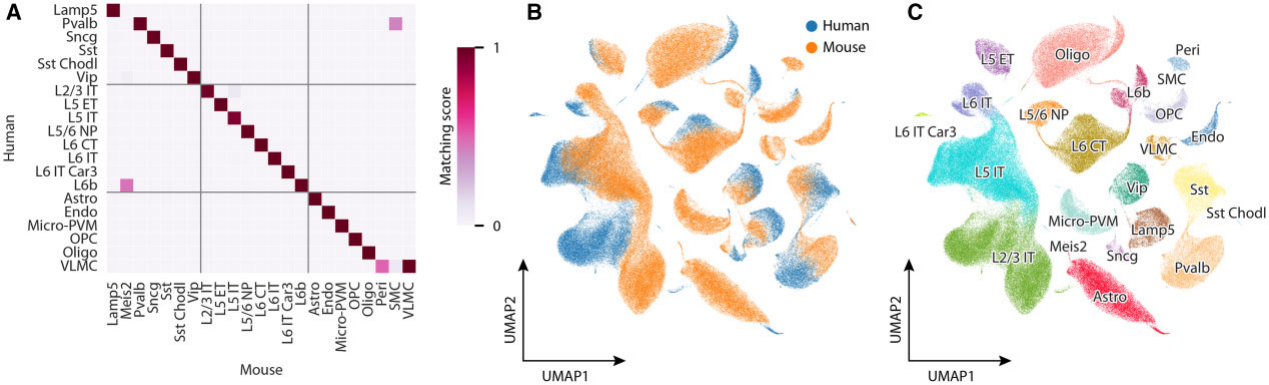
TACTiCS’ performance when matching human and mouse cell types at the subclass resolution. (A) Average confusion matrix of transferred cell types. (B) UMAP of cell embeddings, colored by species. (C) UMAP of cell embeddings, colored by cell type.

Now that we have seen that ProtBERT matches can be a powerful way to capture gene relationships, we use them in TACTiCS to match cell types in mouse and human cortex data. We use the Allen Brain Data, since the cell types have been carefully matched and harmonized by curators. We train TACTiCS for the human–mouse comparison for both the subclass and cross-species resolution. At the subclass resolution, TACTiCS returns the correct cell type for all 23 cell types that are present in both human and mouse (Fig. 4A). The species-specific cell types, mouse Meis2, Peri, and SMC, do not have a one-to-one match with a human cell type. Mouse Peri only matches human VLMC with a score of 0.5, but human VLMC matches mouse VLMC with a higher score of 1.0. Cell types present in both species have matching scores of ≥0.9 while wrong matches all have matching scores ≤ 0.5.

To get better insight into TACTiCS performance, we visualized the 32-dimensional cell embeddings using UMAP (Fig. 4B and C; Supplementary Fig. S3). Individual human and mouse cells do not mix well in the embedding space, but the UMAP does seem to align at the cell type level, i.e. corresponding cell types either overlap partially in the embedding space, or are relatively close. For example, Vip cells form a large cluster with partly human and mouse cells separated, and cells of mixed origin in the middle. The Sncg cells also form a larger cluster, but the separation between the human and mouse cells is more visible. The oligodendrocytes form two separate clusters, but they are closer to each other than to other cell types. The cell type proportions do seem to have an effect on the alignment in the embedding space. Cell types with a similar number of cells in human and mouse, such as Vip (6% in human and 2% in mouse), are clustered more coherently. Cell types with a large difference of occurrence within human and mouse, such as Astro (1% in human and 11% in mouse), form one small distinct cluster that is close to the larger cluster. The mouse-specific cell types Meis2, Peri, and SMC are (correctly) clustered separately from the human cells. Thus, the embedding space can align the cell types across the species, but not the individual cells. Note that this can be due to unresolved batch effects or actual biological differences between the two species.

**Figure 5.**
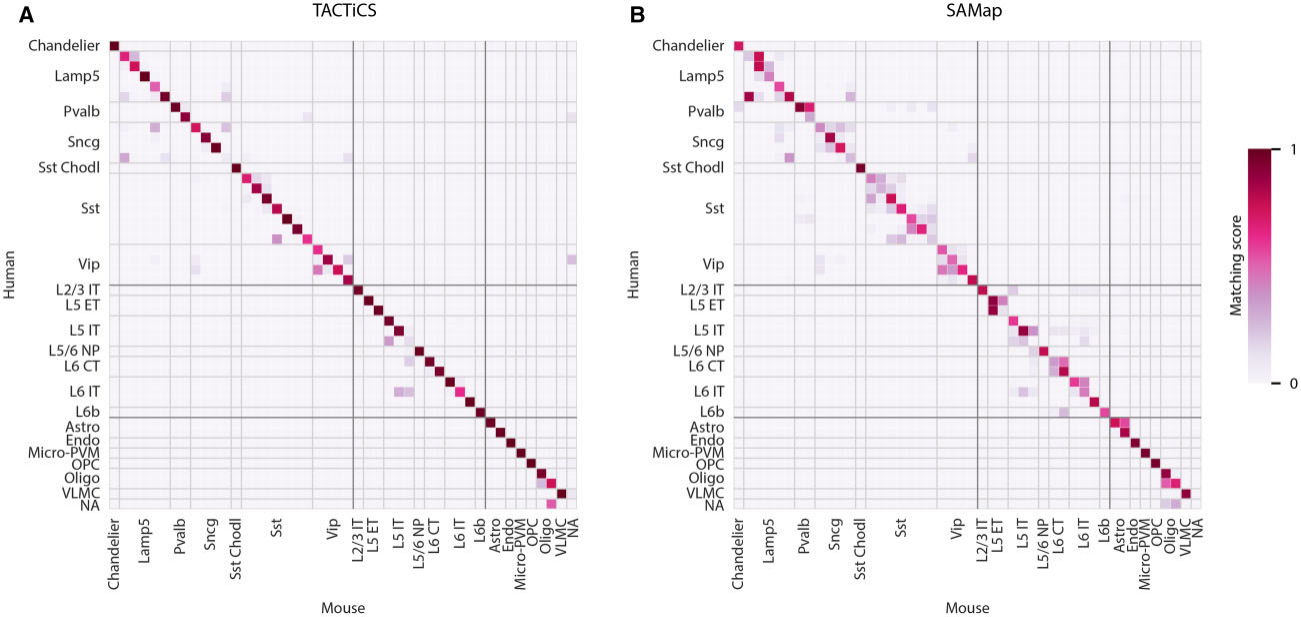
Performance of (A) TACTiCS and (B) SAMap on when matching human and mouse cell types at cross-species resolution. Cross-species cell types are grouped per subclass (indicated with the light-gray lines) and class (indicated with dark-gray lines).

At the cross-species resolution, TACTiCS returns correct matches for the majority of cell types, with a recall of 0.96 (Fig. 5A; Supplementary Fig. S1). The two cell types that are not properly matched, namely a L5-IT subtype and a Sncg subtype, are still matched with closely related cell types. The L5-IT subtype is matched with another L5-IT subtype and the Sncg subtype is matched to a subtype from the similar Lamp5 subclass.

To evaluate the performance of TACTiCS across species with variable evolutionary distance, we tested TACTiCS on cortical cell types between human–marmoset and mouse–marmoset (Table 1). At the subclass resolution, TACTiCS performs similar on all three comparisons with a recall of 1.0. At the cross-species resolution, TACTiCS performs best for the human–marmoset comparison and worst for the mouse–marmoset comparison. These results indicate that the performance of TACTiCS is dependent on the evolutionary distance between the species, since the evolutionary distance to the closest common ancestors from human and marmoset (∼40 mya) is a lot less than human and mouse (∼70 mya).

**Table 1.** ADS and recall for TACTiCS, Seurat, and SAMap on human, mouse, and marmoset.

Comparison	Method	Matching	Subclass		Cross-species	
			ADS	Recall	ADS	Recall
Hu–mo	TACTiCS	P (m:m)	0.991	1.000	0.856	0.956
Hu–mo	TACTiCS	B (m:m)	0.915	0.900	0.509	0.489
Hu–mo	TACTiCS	B (1:1)	0.992	1.000	0.724	0.778
Hu–mo	Seurat	B (1:1)	0.821	0.850	0.435	0.400
Hu–mo (50k)	TACTiCS	P (m:m)	0.894	0.900	0.780	0.822
Hu–mo (50k)	SAMap	P (m:m)	0.814	1.000	0.635	0.733
Hu–mo (50k)	SAMap	B (m:m)	0.827	1.000	0.630	0.800
_____________________________________						
Hu–ma	TACTiCS	P (m:m)	0.981	1.000	0.920	0.956
Hu–ma	TACTiCS	B (m:m)	0.891	0.900	0.848	0.889
Hu–ma	TACTiCS	B (1:1)	0.983	1.000	0.919	0.956
Hu–ma	Seurat	B (1:1)	0.906	1.000	0.697	0.822
Hu–ma (50k)	TACTiCS	P (m:m)	0.982	1.000	0.949	1.000
Hu–ma (50k)	SAMap	P (m:m)	0.892	1.000	0.816	0.978
Hu–ma (50k)	SAMap	B (m:m)	0.899	1.000	0.816	0.978
_____________________________________						
Mo–ma	TACTiCS	P (m:m)	0.990	1.000	0.735	0.733
Mo–ma	TACTiCS	B (m:m)	0.844	0.864	0.483	0.467
Mo–ma	TACTiCS	B (1:1)	0.991	1.000	0.770	0.778
Mo–ma	Seurat	B (1:1)	0.819	0.864	0.488	0.489
Mo–ma (50k)	TACTiCS	P (m:m)	0.928	0.909	0.730	0.733
Mo–ma (50k)	SAMap	P (m:m)	0.798	0.955	0.608	0.689
Mo–ma (50k)	SAMap	B (m:m)	0.823	0.955	0.637	0.689

The gene–gene matching is either done using ProtBERT (P) or BLAST (B) and can be one-to-one (1:1) or many-to-many (m:m).

### 3.3 TACTiCS outperforms SAMap and Seurat in matching cortical cell types across mouse, human, and marmoset

To benchmark TACTiCS, we compare its performance to SAMap and Seurat using three pair-wise comparisons (human–mouse, human–marmoset, and mouse–marmoset). Across all comparisons, TACTiCS has a higher ADS and recall than SAMap and Seurat at the subclass resolution (Table 1). TACTiCS and SAMap perform well for all comparisons with a recall ≥0.95. Seurat performs well for the human–marmoset comparison, but the performance drops for the other two comparisons with a recall of 0.85 and 0.86 for the human–mouse and mouse–marmoset comparisons, respectively. Although the resulting matches of TACTiCS and SAMap are similar, the scores assigned by TACTiCS to those correct matches are higher than SAMap. For instance, SAMap correctly matches human L6b to mouse L6b, but with a very low matching score equal to 0.47, while TACTiCS matches the same cell types with a matching score of 1.0. Interestingly, for the species-specific cell types, TACTiCS suggests matches that have a low score (0.04–0.5), allowing to detect the species-specific cell types. The performance of SAMap and Seurat for the species-specific cell types is not consistent across all cell types and comparisons. For example, SAMap correctly assigns zero scores to mouse Meis2, Peri, and SMC in the human–mouse comparison, but incorrectly matches mouse SMC to marmoset Peri with a high matching score. Likewise, Seurat correctly assigns low scores to mouse Meis2 across all three comparisons, but incorrectly assigns higher scores to mouse Peri and SMC.

At the cross-species resolution, the performance of all methods drops compared with the subclass level as expected, but the difference between the three methods becomes more apparent (Fig. 5; Supplementary Fig. S2). TACTiCS achieved the highest ADS and recall for the human–mouse and mouse–marmoset comparisons. SAMap has a higher recall than TACTiCS for the human–marmoset comparison, but not a better ADS. Seurat performs the worst across all three comparisons and achieves a recall of only 0.4 for the human–mouse comparison. For mismatches between subtypes, TACTiCS usually matches to subtypes within the same subclass, while SAMap regularly maps to cell types from another subclass. While both TACTiCS and SAMap partly match human Sncg to mouse Lamp5, SAMap additionally shows similarity between human Sncg and mouse Vip.

While the human and mouse cells did not overlap much in the UMAP of TACTiCS, Seurat consistently maps the query dataset onto the reference dataset (Supplementary Figs S3 and S4). However, the query dataset is not mapped equally onto the reference dataset, which leaves large regions of the clusters consisting of only one species. For both methods, the mixing of species is the highest for the human–marmoset comparison and lowest for the human–mouse comparison.

To account for the differences in dataset size, we compare TACTiCS and SAMap on the same 50k subset. The performance of TACTiCS drops on the subset compared with the full dataset and does not match all common cell types correctly anymore at the subclass resolution. However, TACTiCS still outperforms SAMap at the higher resolution across all three comparisons.

### 3.4 Using ProtBERT matches improves the cell type matching for TACTiCS

Finally, we assessed the importance of using the ProtBERT embeddings to match genes compared with using BLAST on the final cell type matches. To this end, we trained TACTiCS based on the BLAST many-to-many matches and SAMap using the ProtBERT matches on the human–mouse data. For a fair comparison of ProtBERT to BLAST in SAMap, we only apply the embedding distance threshold to the ProtBERT matches, rather than filtering the gene matches thoroughly. Training TACTiCS at the cross-species resolution using the BLAST matches decreased the ADS and recall by a lot across all comparisons (Table 1). For SAMap, the performance remained similar, except for the human–mouse comparison where the recall decreased from 0.8 to 0.73 when ProtBERT matches were used instead of the BLAST matches.

Additionally, we trained TACTiCS on the BLAST one-to-one matches. At the subclass resolution, the ADS and recall remain similar if BLAST one-to-one is used instead of ProtBERT many-to-many. This is not the case for all comparisons at the cross-species resolution. The performance decreases for human–mouse, remains similar for human–marmoset, and is increased for mouse-marmoset when BLAST one-to-one is used.

## 4 Discussion

Here, we present TACTiCS, a method to accurately match cell types from scRNA-seq data across species. We applied TACTiCS to match cell types across human, marmoset, and mouse motor cortex, species with different evolutionary distances to each other. Even though TACTiCS matches cell types from all three species with high confidence, we showed that human and marmoset cell types are considerably easier to match which correlates with their closer evolutionary distance. Furthermore, we showed that TACTiCS outperforms the state-of-the-art method SAMap on all comparisons with the biggest difference at a higher resolution in favor of TACTiCS. We should note that our evaluation is limited to using only three datasets from one tissue with a relatively small evolutionary distance, while SAMap was originally developed to match cell types across larger evolutionary distances (Tarashansky et al. 2021).

Even though TACTiCS outperforms SAMap on the (finer) cross-species resolution, its performance drops as well. We would like to note that the cell types at this resolution were established by Bakken et al. (2021) by integrating datasets from the different species and clustering them in an embedding space. This resulted in ambiguous clusters which were resolved manually by the authors to determine which cell types would be in one cross-species group. Since these matches are not perfect, it makes sense that we cannot achieve a perfect performance either.

Furthermore, the ground-truth matches used for evaluation are based on analyses performed using BLAST one-to-one matches, also causing unwanted differences when comparing results. This might explain why the performance of TACTiCS using BLAST one-to-one is comparable to using ProtBERT many-to-many matches. Here, we only see an improvement for species with larger evolutionary distances (i.e. human–mouse comparison).

All the results obtained by TACTiCS were obtained using the same hyperparameters, which have not been tuned. Although tuning the hyperparameters could potentially improve matches between species, the advantage of the current set of hyperparameters is that they show robust performance across all pairwise comparisons regardless of species and resolution (i.e. subclass or cross-species).

Gene matching is one of the main components of TACTiCS. We match genes based on the distance between their corresponding protein embeddings, which are generated using ProtBERT instead of the commonly used sequence similarity based on BLAST. Even though the top-1 matches of ProtBERT and BLAST are largely similar, we have shown that using ProtBERT instead of BLAST distances improves the performance of TACTiCS. When aligning sequences using BLAST, every amino acid is considered to be equally important, while we speculate that ProtBERT focuses more on functional domains. During further research, it would be interesting to dive deeper into the ProtBERT embedding space and see how this could be used to learn more about the relationships between cell types and the genes involved. A downside, however, of using ProtBERT distances is that the protein sequence is needed and as a consequence, we can only use coding genes. Using DNA sequence embedding models, e.g. DNABert (Ji et al. 2021), for non-coding genes, could in the future be used to overcome this limitation.

Some cell types, such as Meis2 and Peri in mice, are species-specific. A limitation of our current approach is that the classifiers we built in TACTiCS are missing a rejection option and therefore we cannot identify these species-specific cells automatically. Although we observed that TACTiCS usually assigns a low matching score to these species-specific cell types, it is, however, important to realize that the matching score represents the average accuracy of the two classifiers and does not represent an absolute measure of cell type similarity. For instance, if two human cell types are very similar, predictions for a mouse cell type may be split over these two human cell types (e.g. both get a score of 0.5). This is, for instance, the case with the Vip cross-species clusters in Fig. 5A. This lower score indicates that there are similar human cell types in the data that both look like this mouse cell type. A high score, however, does not guarantee that the two cell types are very similar. It only indicates that these two cell types are most similar to each other and that they are transcriptionally very distinct from the other cell types in the dataset. In other words, the scores are summaries of the classification results, and as such, they are very much dependent on the cell types present in both datasets (i.e. the scores will change if one cell type is missing from one of the two species).

When inspecting the cell embeddings in the low dimensional space, we notice that the cells from difference species are not well mixed. Matching cell types, however, are closest to each other and species-specific cell types are more separated from all other cells. There are many data integration methods developed for single-cell data, such as scVI (Lopez et al. 2018), that would achieve a significantly better integration. Since data integration is not the main goal of TACTiCS, we did not add an explicit mixing component to the loss function. The current loss function enforces that neighboring cells from the other species can predict the other cell’s gene expression profile. This enforces cells of the same cell type to be the closest, but not to fully overlap. Adding a component to the loss that forces cells to be mixed (e.g. to have neighbors of both species) could greatly improve the integration. Alternatively, if good integration is a user’s desire, an option would be to replace the component of TACTiCS that generates the cell embeddings with another data integration method such as scVI. The flexible architecture of TACTiCS allows the individual components (gene matching, cell embedding, and cell classification) to be easily replaced, extended, or integrated with different methods.

With TACTiCS, we showed that using protein embeddings to match genes is a viable alternative to BLAST when matching cell types based on their scRNA expression levels across species. TACTiCS can accurately match cell types at different resolutions for large datasets, outperforming Seurat and SAMap. We envision that this fast and accurate cell type matching method will make comparative analyses across species considerably easier, contributing to, e.g. to the study of cell type evolution or translational research.

## Supplementary Material

btad248_Supplementary_Data
