# Supplementary material for: Cell type matching across species using protein embeddings and transfer learning
Source: Bioinformatics. 2023 Jun 30;39(Suppl 1):i404–12. doi: 10.1093/bioinformatics/btad248 (PMC10311290; doi:10.1093/bioinformatics/btad248)
Supplement: btad248_Supplementary_Data [file btad248_supplementary_data.pdf]

Supplementary

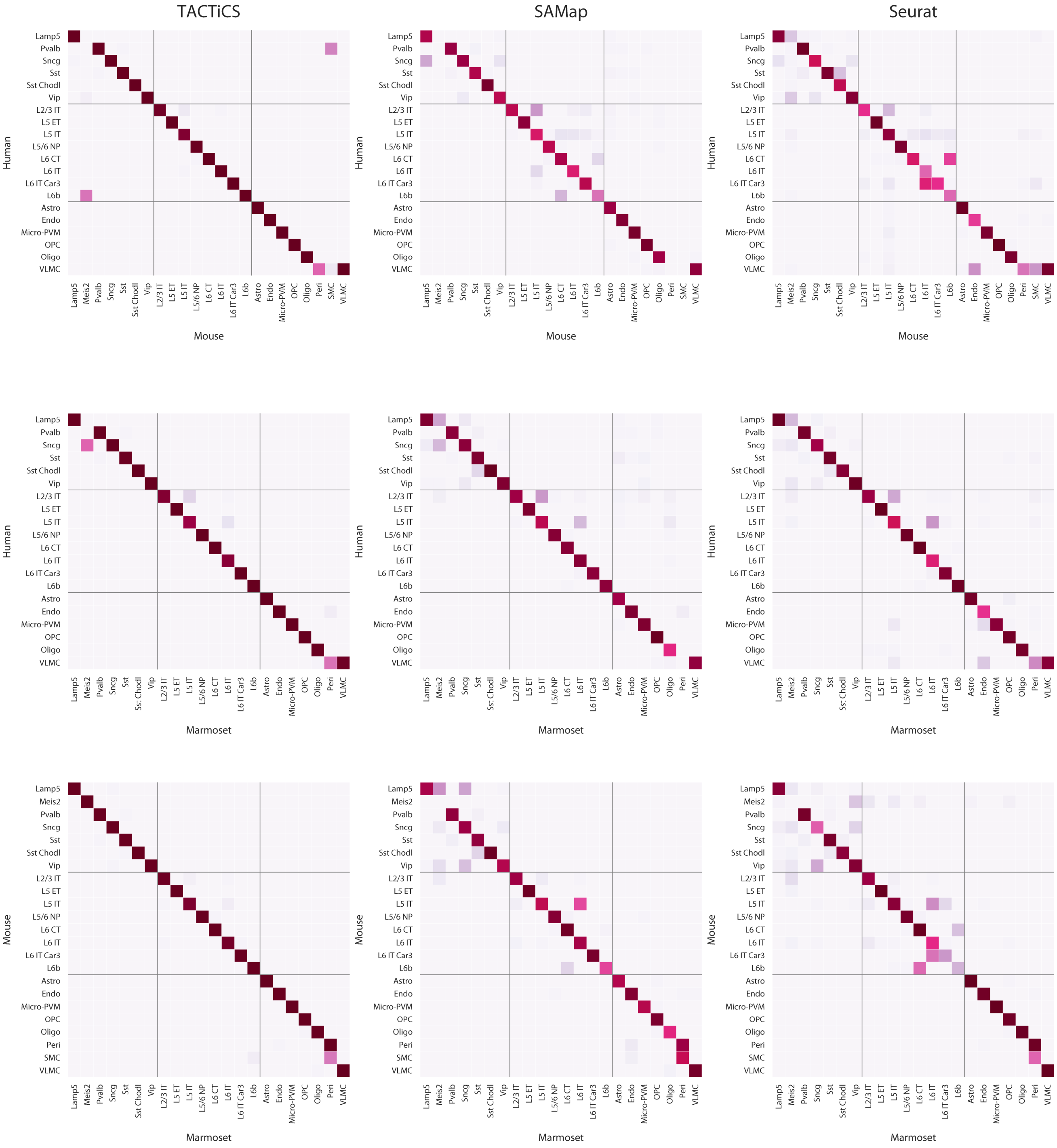

Fig. S1: Performance of TACTiCS, SAMap and Seurat on three pairwise comparisons (human-mouse, human-marmoset, and mouse-marmoset) on the subclass resolution, using Prot-BERT many-to-many matches for TACTiCS, BLAST many-to-many matches for SAMap and BLAST one-to-one matches for Seurat.

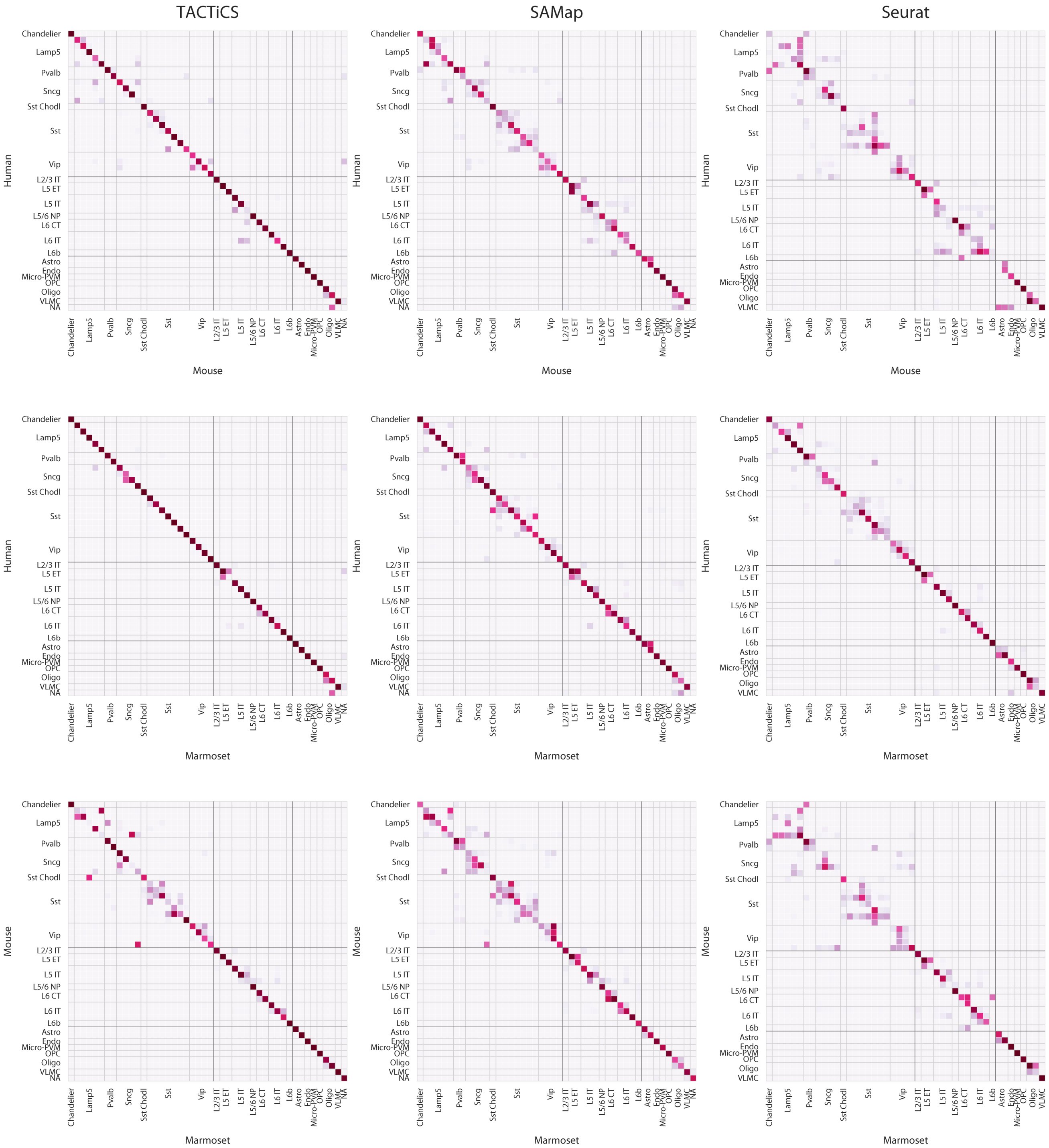

Fig. S2: Performance of TACTiCS, SAMap and Seurat on three pairwise comparisons (human-mouse, human-marmoset, and mouse-marmoset) on the cross-species resolution, using ProtBERT many-to-many matches for TACTiCS, BLAST many-to-many matches for SAMap and BLAST one-to-one matches for Seurat.

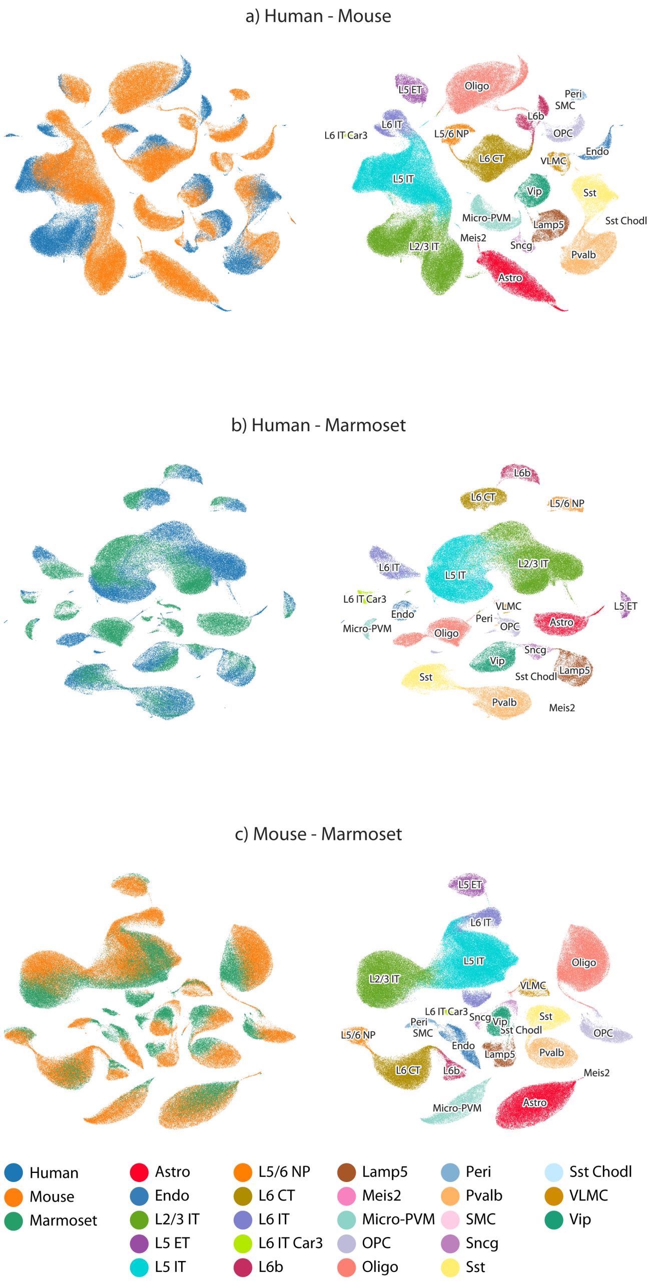

Fig. S3: Uniform manifold approximation and projection (UMAP) dimensional reduction of cell embeddings by TACTiCS for three pairwise comparisons (human-mouse, human-marmoset, and mouse-marmoset) colored by species (left), and cell type (right).

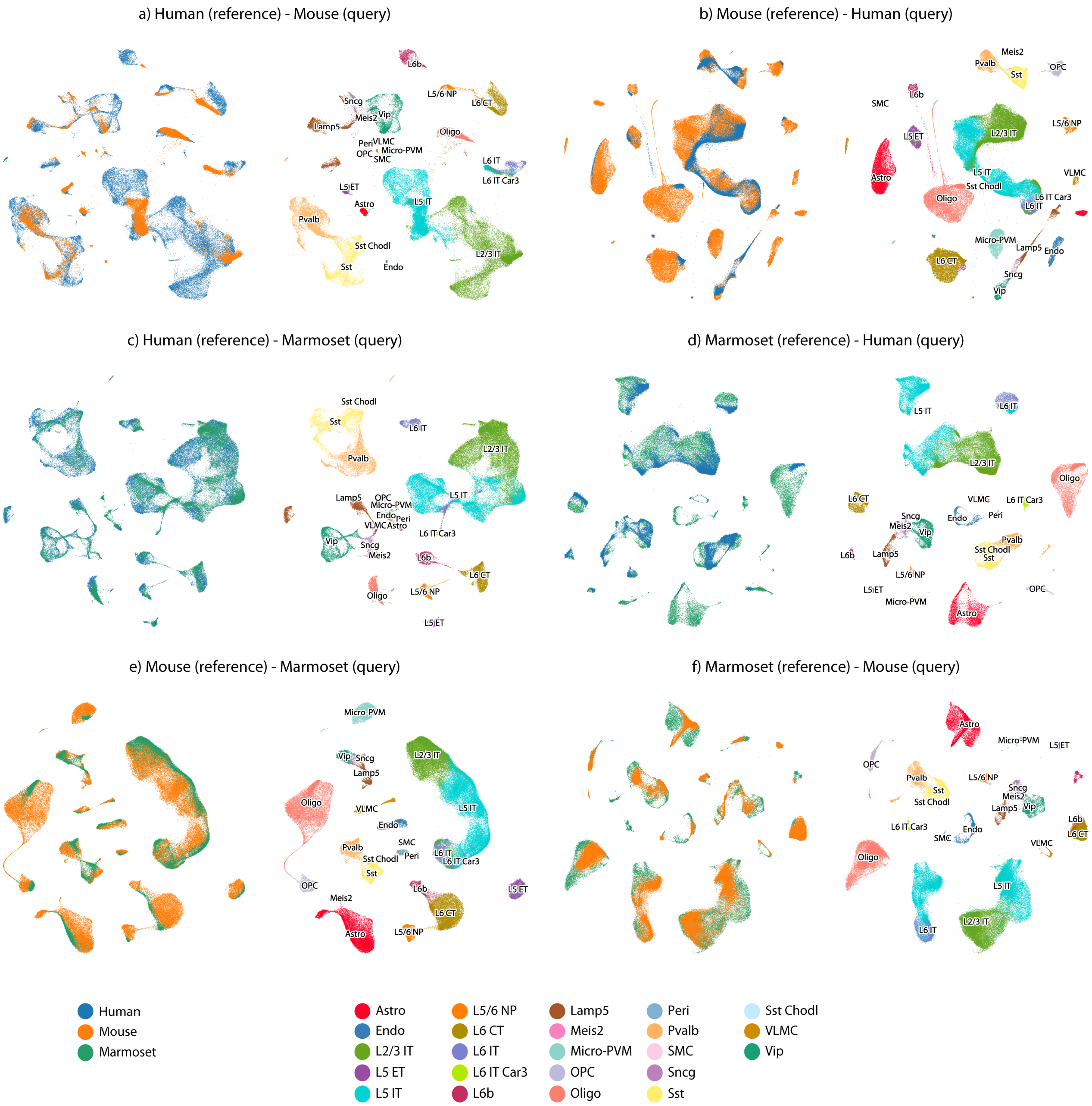

Fig. S4: Uniform manifold approximation and projection (UMAP) dimensional reduction of integrated data by Seurat for three pairwise comparisons (human-mouse, human-marmoset, and mouse-marmoset) colored by species (left), and cell type (right).
